# Comments on “Quantifiable urine glyphosate levels detected in 99% of the French population, with higher values in men, in younger people, and in farmers”

**DOI:** 10.1007/s11356-022-20860-4

**Published:** 2022-05-20

**Authors:** William Reeves, John L. Vicini, John T. Swarthout, Bruce M. Young, Pamela K. Jensen

**Affiliations:** Bayer Crop Science, 700 Chesterfield Parkway West, Chesterfield, MO 63017 USA

**Keywords:** Glyphosate, France, General population, Dietary habits, Occupational exposure

To the Editor,

Grau et al. reported quantifiable levels of glyphosate in 99.8% of urine samples collected in France between 2018 and 2020. Conclusions about these data rest on the reliability of the analytical method and the ability to put the data into the context of safety standards. Rather than demonstrating evidence of a health concern for the French population, data from the study confirm that human exposures to glyphosate are well below safety thresholds established by regulatory authorities in Europe.

Grau et al. relied on an ELISA method to estimate glyphosate concentrations in urine samples. This method was designed for testing glyphosate in water and is meant to serve as a screening tool to identify samples for more robust quantitative analysis. The ELISA does not provide a clear confirmation of a specific concentration (Vicini et al. 2021)., and as the authors admit, tends to overestimate glyphosate levels in urine.

Additionally, the publication is missing key information that would have helped readers understand whether the reported values indicate a health concern. Other publications presenting human urinary glyphosate data provide estimates of daily exposure and compare those estimates to allowable exposure levels (Niemann et al. 2015; Solomon 2020; Vicini et al. 2021). These comparisons consistently demonstrate that human exposures to glyphosate are well below established safety thresholds.

Grau et al. reported the highest mean urinary glyphosate concentration was 2.05 ± 1.29 ng/ml for study participants under the age of 16 years. Taking this largest mean value plus three times the standard deviation gives an estimated upper end concentration of 5.92 ng/ml. Assuming a 30-kg child with a 1-L/day urinary volume results in an estimated intake of 0.000987 mg/kg-body weight/day or 0.2% of allowable exposures in the European Union (currently 0.5 mg/kg/day). This value is based on 20% bioavailability (EFSA 2015). Even using the 1% bioavailability value Grau et al. cite, the estimated exposure corresponding to the highest urinary concentration would still be 4.0% of allowable EU exposures for glyphosate.

It is important to note that Grau et al. did not disclose that the sponsor of their study and the employer of three authors, Association Campagne Glyphosate, advocates for a ban on the use of glyphosate-based herbicides in France. This information would have provided readers with important context regarding the purpose of the study and publication. This letter was drafted by scientists employed by Bayer Crop Science, a manufacturer of glyphosate. The method used to calculate exposure was first published by Niemann et al. (2015).
